# Consumption of dopamine receptor 1 agonist SKF-38393 reduces constant-light-induced hyperactivity, depression-like, and anxiety-like behaviors in a sex specific manner in C57BL/6J mice

**DOI:** 10.3389/fnbeh.2025.1537048

**Published:** 2025-03-12

**Authors:** Grace E. Guindon, Alexis Anzalone, Samantha G. Burke, Cloey A. Murphy, Maria E. Milano, John C. Price, Stephanie Tadros, Alexander T. McFarland, Fernanda Medieros Contini, Joseph A. Seggio

**Affiliations:** Department of Biological Sciences, Bridgewater State University, Bridgewater, MA, United States

**Keywords:** light, circadian rhythm, anhedonia, dopamine, open field (OF), mouse behavior

## Abstract

Artificial light exposure during nighttime, including constant light (LL), is an increasingly prevalent environmental occurrence linked to impaired mood and cognitive impairments in both humans and animal models. Dopamine and dopamine 1 receptors are well known to modulate circadian rhythms and mood. This study investigated the effects of LL on anxiety-like, depressive-like, and cognitive behaviors in male and female C57BL/6J mice and assessed whether consumption of SKF-38393, a dopamine 1 receptor agonist, can mitigate these negative behavioral outcomes. Mice were exposed to LL or a standard 12:12 light:dark cycle (LD) for 6 weeks, with subgroups receiving either SKF-38393 or water. All mice had their circadian rhythms continuously monitored and were placed within behavioral tests that assayed their anxiety-like, depressive-like, and learning and memory behaviors. Behavioral assays revealed that LL increased hyperactivity and anxiety-like behaviors, which were mitigated by SKF-38393 consumption in both sexes. In addition, male mice exhibited anhedonia under LL, which was alleviated by SKF-38393, whereas female mice were resistant to LL-induced anhedonia. Sex differences emerged in fluid consumption independent of lighting condition, with females consuming more SKF-38393, and in responses to DA on behavior, including novel object recognition and exploration. These results indicate that low dose oral consumption of dopamine 1 receptor agonists can ameliorate some of the negative behavioral effects of LL exposure. This study highlights the complex interplay between chronic light, dopamine, and sex in influencing mood and behavior, suggesting potential modulatory roles for dopamine 1 receptor agonists in regulating behavioral outcomes to circadian disturbances.

## Introduction

Light-at-night and chronic light exposure, whether dim or bright, is an increasingly pervasive environmental factor due to urbanization and artificial lighting. Studies using rodent models have demonstrated that dim light-at-night, bright light-at-night, and constant light (LL) can induce anxiety-like and depressive-like behaviors, as well as impair learning and memory (Copenhaver et al., 2022). Similarly, human studies have identified links between light-at-night and mood disorders, suggesting a translational relevance for investigating the neurobiological mechanisms underlying these effects (Guindon et al., 2024). The increasing use of electronic devices, including TVs, smartphones, and tablets at night has also been linked to poorer mood in humans (Figueiro and Overington, 2016). These findings emphasize the importance of understanding the specific pathways through which chronic and evening light exposure influences behavioral outcomes.

Dopamine (DA), a key neurotransmitter involved in mood regulation and reward processing, has emerged as a potential modulator of light-induced behavioral changes. DA signaling plays a crucial role in regulating circadian rhythms, with evidence showing that activation of D1-like dopamine receptors (D1R) can influence the expression of “core clock” genes in brain regions which govern mood and reward responses (Imbesi et al., 2009). Additionally, D1Rs are present in the mammalian suprachiasmatic nucleus, the brain area that regulates circadian rhythmicity (Rivkees and Lachowicz, 1997) and are necessary for circadian entrainment (Grippo et al., 2017). Dopamine concentration and release fluctuates with circadian periodicity in both the striatum and nucleus accumbens, areas that control behavioral responses and the reward pathway (Castaneda et al., 2004). Depletion of DA and DRs disrupt both the amplitude and rhythmicity of sleep–wake cycles, indicating dopamine’s participation in the bodies oscillating rhythms (Fifel et al., 2014). Furthermore, the presence of extracellular dopamine has been seen to play a role in regulating clock genes, including *period1* and *period2*, in tissues peripheral to the suprachiasmatic nucleus (the central oscillator) (Hood et al., 2010). There remains a connection between altered dopaminergic signaling and circadian disruptions contributing to adverse behavioral consequences. Additionally, D1Rs are implicated in the regulation of anxiety-like behaviors (Tong et al., 2023) and activation can potentiate the effects of antidepressants (Shuto et al., 2020). The capacity of DA to modulate mood and circadian rhythmicity further supports its potential as a target for modulating the behavioral outcomes of aberrant light exposure. This study investigates the modulatory effects of oral SKF-38393, a selective D1R agonist, on anxiety-like, depressive-like, and cognitive behaviors in response to LL in mice.

## Methods

### Animals

A total of 32 male (M) and 32 female (F) C57BL/6J (B6 - #000664) mice were purchased from The Jackson Laboratory (Bar Harbor, ME, USA) at approximately 8-weeks of age. All animals were initially placed into circadian home-cage activity monitoring cages as previously described (Nascimento et al., 2016), under a 12:12 light:dark (LD) cycle (0600–1800 light), approximately 150 lux, consuming regular chow (RC, LabDiet5001, St. Louis, MO, USA) and water *ad libitum*, for 2 weeks of acclimation. Afterwards, half of each sex were placed into constant light (LL) [150 lux, starting at Zeitgeber Time (ZT) 12 on the last day of LD, additional lighting specifics (Burns et al., 2024)], while the remaining stayed in the LD cycle; additionally, half of each group in LD and LL were given 24-h access to a single bottle of 10 μg/mL solution of SKF-38393 (DA—Millapore-Sigma-Aldrich, St. Louis, MO, USA) dissolved in drinking water while the remaining received a single bottle of water (W) (both solutions *ad libitum*, 150 mL bottle, new fluid provided every 5–7 days as needed). This dose was chosen because it is a safe but effective dose to induce behavioral changes in a previous study (Huang et al., 2014). As such, there were eight groups of *n* = 8: (1) M/W/LD, (2) M/DA/LD, (3) F/W/LD, (4) F/DA/LD, (5) M/W/LL, (6) M/DA/LL, (7) F/W/LL, and (8) F/DA/LL. Fluid consumption was measured manually by pouring the liquid into a graduated cylinder weekly (Friday) between ZT/CT (Circadian Time) 4–8. These measurements were done in conjunction with an empty cage with bottles of the same amount of liquid which was also measured to account for spillage during animal maintenance; we did not use an automatic measurement system to track fluid consumption which not only would measure consumption but also the timing of mouse drinking.

### Behavioral assays

**Figure 1:**
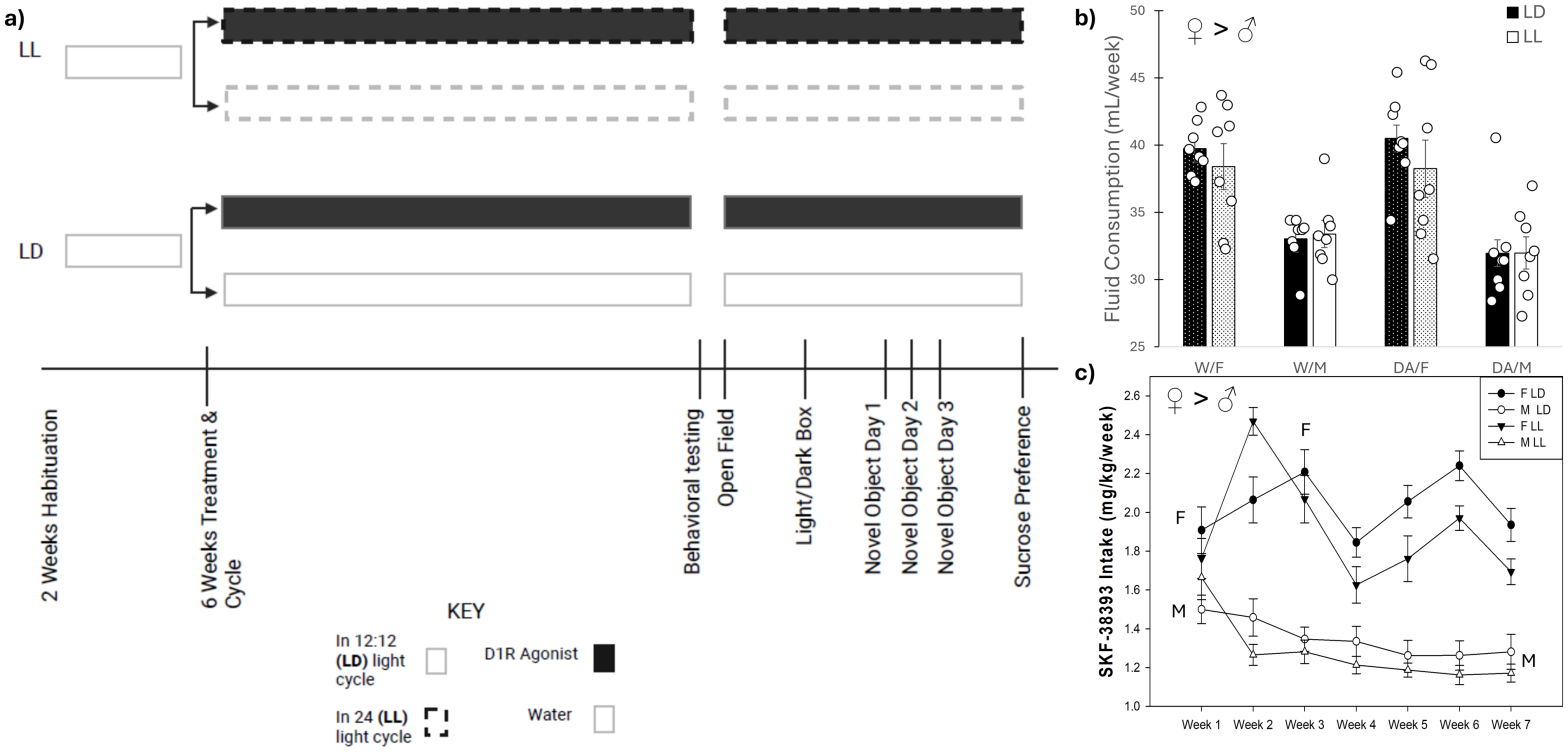
Timeline of behavioral assays and fluid consumption. **(a)** Schematic diagram. The experimental setup of the groups of mice for both sexes and a timeline (with order) of the behavioral assessment. **(b)** Average weekly fluid consumption. Weekly consumption was averaged across the 7 weeks. Female mice consumed more fluid, both water and DA, compared to males. **(c)** Weekly SKF-38393 intake. Female mice exhibited increased oral dosing of DA compared to males across all weeks. Female mice increased their DA consumption during the second and third weeks, compared to the first week on the compound. Male mice reduced their DA consumption after the first week for the remainder of the experiment. Mean ± SEM. ♀ > ♂: indicates sex difference; M ➔ M: indicates significant decrease in DA dosing; F ➔ F: indicates significant increased DA dosing, at *p* ≤ 0.050.

All of the following behavioral assays described below were conducted during the middle of each animal’s inactive time (approximately ZT 6 for LD animals or CT 6 for LL animals) in 250 lux. ZT/CT 6 was selected because: (a) the LD animals were exposed to several hours of light prior to the behavioral assays, (b) the assays were conducted in the light for all animals, and (c) for the alignment of activity phases for each animal (i.e., all animals were tested during the middle of their inactive time). Each different behavioral assay listed below was conducted in the order listed below and separated by at least 3 days to allow for recovery (Paylor et al., 2006) and continued with their treatments during the interim (Figure 1a).

After 6 weeks of exposure to their respective lighting and treatment, each animal was placed into open field and light–dark box assays, using the automated tracking SmartCage System (AfaSci Inc., Redwood City, CA, USA, Xie et al., 2012; previously described Hicks et al., 2016). For the open field, the time spent in the center zone, rears, distance, and velocity were recorded. For the light–dark box, percent time in the dark zone, first dark zone entry latency, and the number of transitions between the light and dark zones were recorded. Mice were allowed to freely roam both the open field and light–dark box for 10 min. Additionally, all mice were subjected to a 3-day novel object recognition test, similar to a previous study (Capri et al., 2019). On Days 1 and 2, an individual mouse is placed into the arena (19.56 W × 30.92 L cm) with two of the same object (two rectangle Lego™ towers, same color, two blocks high, placed on opposite ends of the field, taped to the bottom) and given 10 min to explore. On Day 3, the mice were placed into the arena again, except this time the right object was replaced with a new object (circular Lego™ tower of a different color, two blocks high, taped to the bottom) and given 5 min to explore. The number of touches/sniffing of at least 1 s for both the left and right objects, as well as the amount of time spent on the right half of the box (regardless of interaction with the object), were manually recorded by four humans under blind conditions, accounting for equal numbers among the groups by a fifth individual. In between the different days, the animal was returned to their home cage and continued with their treatments.

After the completion of the novel object test, a sucrose preference test was performed on all mice. Each mouse was given two bottles *ad* libitum—one bottle of 1% (w/v) sucrose solution and the other bottle plain water for 24 h from ZT/CT 6 to ZT/CT 6. Mice were not deprived of food/fluid before this test to avoid potential or additional withdrawal effects due to removal of SKF-38393 the day prior to the assay. For the DA-consuming mice, the DA bottle was removed and replaced with the sucrose solution bottle. Half of each group had their sucrose bottle on the left side, while the other half of each group had their sucrose bottle on the right side to account for placement preference. After an uninterrupted 24 h, each fluid was measured, and a sucrose preference ratio (sucrose solution/total fluid) was calculated.

### Statistical analyses

Chi-square periodograms and circadian locomotor activity were calculated using Clocklab’s (Actimetrics, Wilmette, IL, USA) batch analysis. Three-way ANOVAs with Tukey post-hoc pairwise comparisons were used to determine mean differences among the groups. If a three-way interaction was significant, the data were further analyzed for each variable using two-way ANOVAs. A two-way repeated measures ANOVA with Bonferroni-corrected post-hoc analyses were conducted to ascertain any changes in SKF-38393 consumption over the course of the 7 weeks (for DA animals only) to determine if these variables changed over time during the course of the experiment.

## Results

### Fluid consumption

Females consumed more weekly liquid compared to males, regardless of fluid type (*F*_1, 56_ = 55.75, *p* < 0.001) (Figure 1b). The two-way repeated measures ANOVA revealed that there were sex differences in mg/kg SKF-38393 consumption over the 7-week timeframe regarding (*F*_6, 168_ = 8.11, *p* < 0.001). Females increased their SKF-38393 consumption during weeks 2 and 3 compared to week 1 (*p* = 0.001 and 0.006, respectively), while males decreased their SKF-38393 consumption after the first week (all *p* < 0.001). Additionally, females consumed more SKF-38393 than males in both lighting conditions (*F*_1, 28_ = 111.06, *p* < 0.001) (Figure 1c).

### Circadian locomotor activity

**Figure 2:**
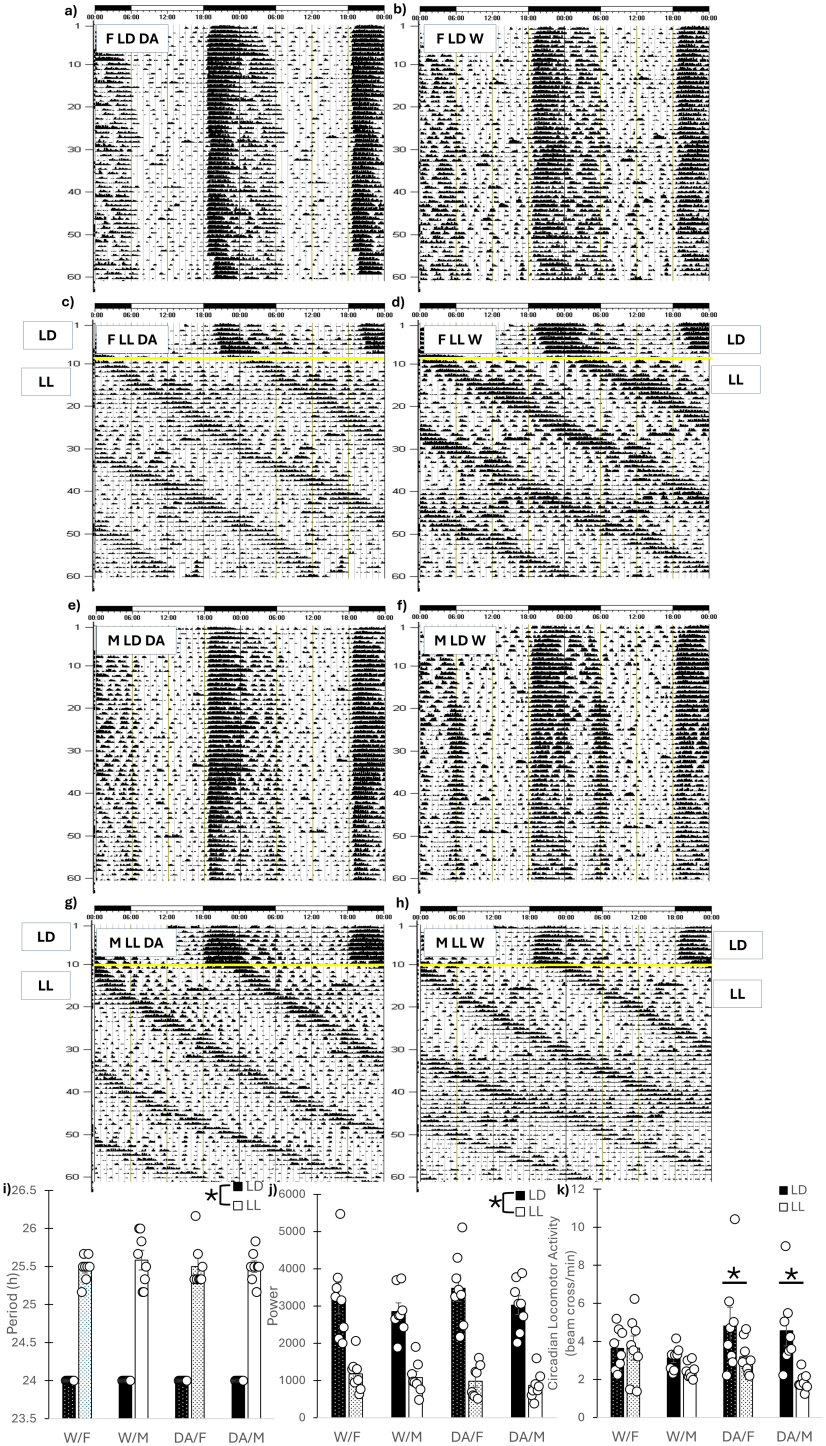
Circadian rhythms. Representative actograms. **(a)** Female LD DA, **(b)** Female LD Water, **(c)** Female LL DA, **(d)** Female LL Water, **(e)** Male LD DA, **(f)** Male LD Water, **(g)** Male LL DA, **(h)** Male LD Water. The yellow line indicates placement into LL. **(i)** Circadian Period. LL lengthened circadian period, but DA consumption had no effects. **(j)** Circadian Power. LL reduced circadian power, but DA consumption had no effects. **(k)** Weekly Circadian Home-cage Locomotor Activity. Weekly activity was averaged across the 7 weeks. For both sexes, consumption of DA led to decreases in circadian activity in LL compared to LD. No differences in locomotor activity was observed for water-consuming animals. Mean ± SEM. *: indicates significant pairwise difference, *p* ≤ 0.050.

Representative actograms are presented in Figures 2a–h. All animals were able to entrain to a LD cycle and all animals in LL remained rhythmic throughout the study. As expected LL lengthened circadian period (*F*_1, 56_ = 36.75, *p* < 0.001) and reduced circadian power (*F*_1, 56_ = 156.29, *p* < 0.001) but no other differences were found (Figures 2i,j). Regarding circadian home-cage locomotor activity, females exhibited more activity than male mice regardless of cycle or fluid (*F*_1, 56_ = 5.08, *p* = 0.028). A cycle x fluid interaction was also uncovered for activity (*F*_1, 56_ = 6.39, *p* = 0.014). DA-consuming mice in LD exhibited increased activity compared to DA-consuming mice in LL (*p* = 0.001) and a non-significant increase compared to W/LD (*p* = 0.061), but no differences were found between water consuming mice (*p* = 0.92) (Figure 2k).

### Behavioral assays

**Figure 3:**
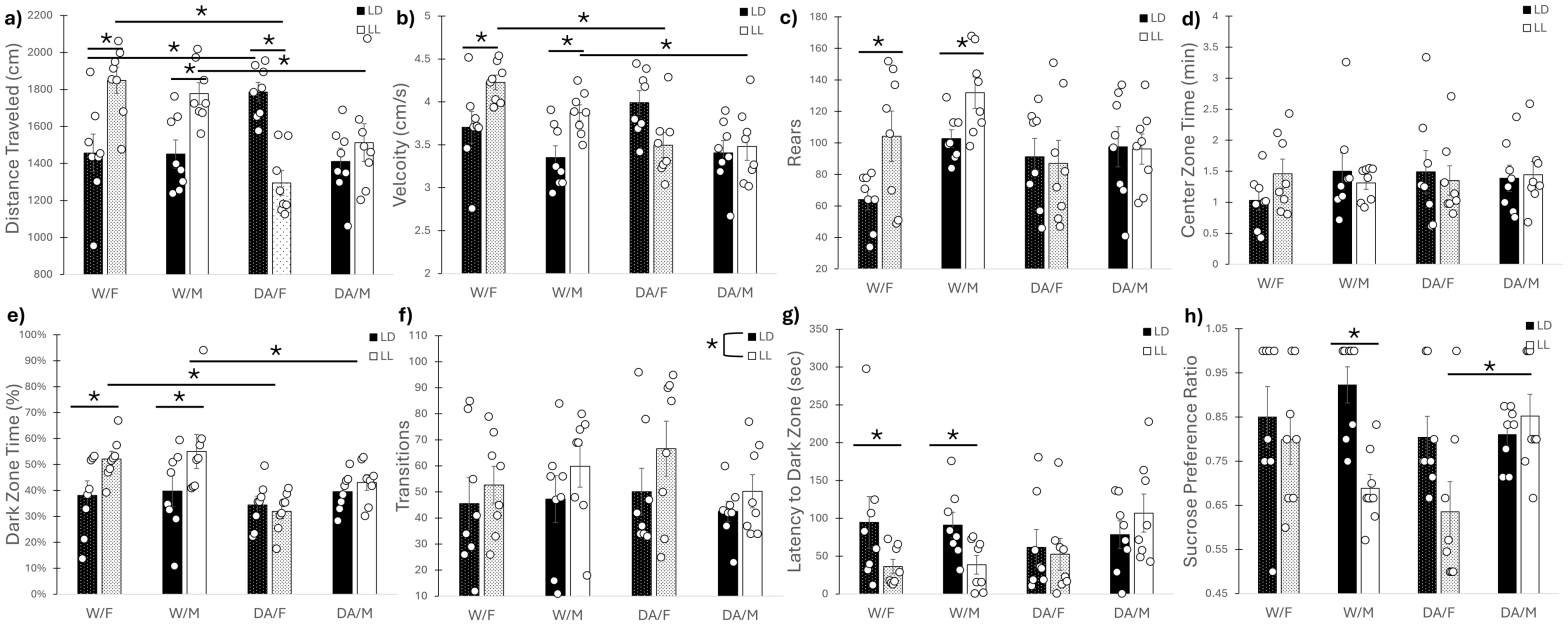
Behavioral assays. **(a)** Distance traveled in open field. LL increased distance traveled for water-consuming mice of both sexes, while DA consumption prevented the LL-induced increase. DA-consuming female mice decreased their distance traveled in LL. **(b)** Velocity in open field. LL increased velocity for water-consuming mice of both sexes, while DA consumption prevented the LL-induced increase. **(c)** Rears in open field. LL increased rearing for water-consuming mice of both sexes, while DA consumption prevented the LL-induced increase. **(d)** Center zone occupancy in open field. No differences were observed. **(e)** Dark zone time in L-D Box. LL mice consuming water exhibited increased time within the dark zone compared to LD/water mice, which was also significantly elevated compared to DA-consuming mice in LL. **(f)** Transitions in L-D box. LL increased the number of transitions between the two zones, but DA consumption had no effects. **(g)** Dark zone latency in L-D box. LL mice consuming water exhibited decreased latency to first dark zone entry, while DA consumption negated this effect. **(h)** Sucrose preference. Male mice, but not female mice, consuming water experienced reduced sucrose preference in LL compared to LD, while male mice consuming DA in LL had no such reduction. Female mice in LL consuming DA exhibited reduced sucrose preference compared to DA-consuming males in LL. Mean ± SEM. *: indicates significant pairwise difference, *p* ≤ 0.050.

A three-way interaction was uncovered for distance traveled in the open-field (*F*_1, 56_ = 10.40, *p* = 0.002) (Figure 3a). For the males, while LL exhibited increased distance traveled compared to LD for water-consuming animals (*p* = 0.045), no differences were found for DA-consuming mice between LD and LL (*p* = 0.97). Meanwhile, DA consumption in LL significantly reduced distance traveled compared to LD for female mice (*p* = 0.001); there was also a difference between LD/F/W and LD/F/DA mice as well (*p* = 0.042). Additionally, a cycle/drink interaction was revealed (*F*_1, 56_ = 29.35, *p* < 0.001), where W/LL increased distance traveled compared to DA/LL animals (*p* = 0.001). Cycle/drink interactions were found for velocity (*F*_1, 56_ = 15.51, *p* < 0.001) (Figure 3b) and rears (*F*_1, 56_ = 6.20, *p* = 0.016) (Figure 3c) in the open-field; whereas no differences were found between DA-consuming animals (*p* = 0.39, *p* = 0.99 for velocity and rears, respectively), water-consuming animals in LL exhibited increased velocity and rears compared to LD animals (*p* = 0.001, *p* = 0.010, respectively). Additionally, DA/LL exhibited reduced velocity compared to W/LL animals of both sexes (*p* = 0.001). While reduced compared to W/LL, DA/LL exhibited no statistically significant difference for rears (*p* = 0.073). No differences were found for center zone time (*F*_1, 56_ = 1.74, *p* = 0.19) (Figure 3d).

Cycle/drink interactions were found for dark zone time in the light–dark box (*F*_1, 56_ = 5.83, *p* = 0.019) (Figure 3e) and for latency to first dark zone entry (*F*_1, 56_ = 5.22, *p* = 0.026) (Figure 3g). Water-consuming animals in LL spent more time in the dark zone compared to both water-consuming animals in LD (*p* = 0.004) and DA-consuming animals in LL (*p* = 0.001). Additionally, LL decreased the latency to the first dark-zone entry for water-consuming animals compared to LD animals (*p* = 0.039), but not for DA-consuming mice (*p* = 0.96). While animals in LL exhibited increased number of transitions between the two zones compared to LD mice (*F*_1, 56_ = 4.05, *p* = 0.049), neither sex (*F*_1, 56_ = 0.47, *p* = 0.45) nor fluid (*F*_1, 56_ = 0.038, *p* = 0.85) had any effects (Figure 3f).

A three-way interaction was revealed for sucrose preference (*F*_1, 56_ = 8.55, *p* = 0.005) (Figure 3h). Subsequent two-way analyses showed that DA-consuming males exhibited increased sucrose preference compared to DA-consuming females in LL (*p* = 0.045). Additionally, water-consuming males in LL exhibited reduced sucrose preference than in LD (*p* = 0.020), while for females, there was no difference (*p* = 0.21). Additionally, a fluid x sex interaction (*F*_1, 56_ = 8.55, *p* = 0.003) for total fluid consumed during the sucrose preference assay was found. Whereas DA-consuming females exhibited increased fluid consumption (both water and 1% sucrose solution) compared to water-consuming females (*p* = 0.001), no differences were found for males (*p* = 0.99), once again highlighting that female mice tend to consume more fluid than males.

**Figure 4:**
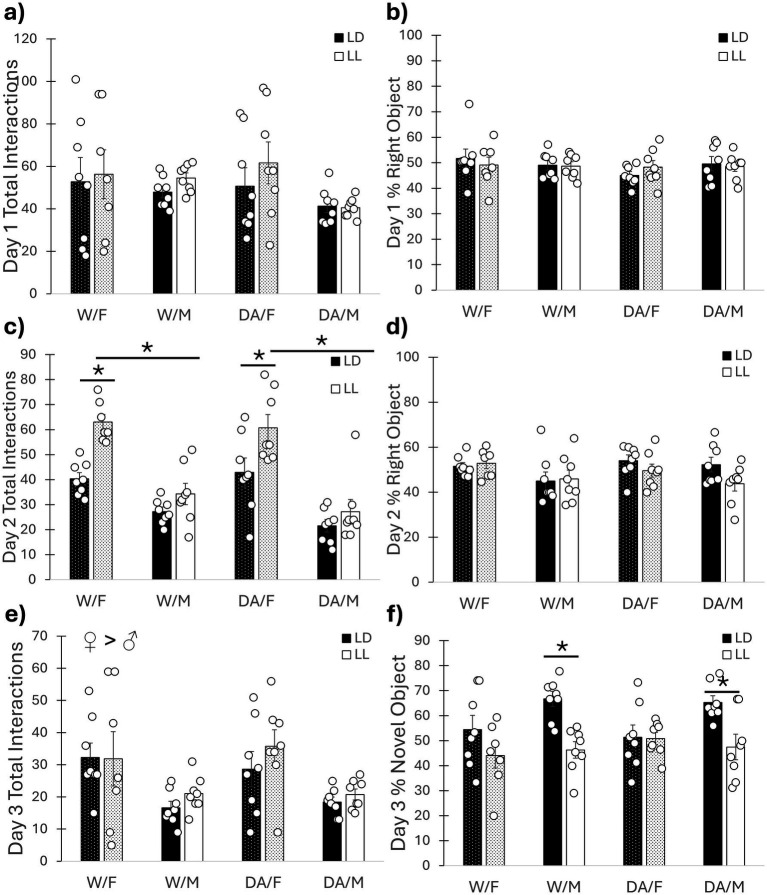
Novel object recognition test. **(a)** Day 1 Object Interactions. No observed differences. **(b)** Day 1 Object Preference. No observed differences. **(c)** Day 2 Object Interactions. Female mice exhibited increased object exploration compared to males. Female mice in LL exhibited increased exploration compared to both males in LL and females in LD, regardless of DA consumption. **(d)** Day 2 Object Preference. No observed differences. **(e)** Day 3 Object Interactions. Female mice had increased exploration compared to males, regardless of DA consumption or lighting condition. **(f)** Day 3 Novel Object Preference. Male mice in LL exhibited reduced exploration of the novel object compared to males in LD, indicating poorer memory. Female mice exhibited no such deficit. DA consumption had no effect on learning and memory for the novel object. Mean ± SEM. ♀ > ♂: indicates sex difference; *: indicates significant pairwise difference, *p* ≤ 0.050.

For the novel object test, no differences were found among the groups total interactions (*F*_1, 56_ = 0.64, *p* = 0.43) with the objects (both the same) as well as preference for one side or the other for Day 1 (*F*_1, 56_ = 0.28, *p* = 0.60) (Figures 4a,b). For Day 2, a cycle/sex interaction was found for total number of object interactions (*F*_1, 56_ = 6.70, *p* = 0.012), where females in LL exhibited increased interactions with objects compared to both females in LD and males in LL (both *p* < 0.001), without affecting preference for either side (*F*_1, 56_ = 0.20, *p* = 0.66) (Figures 4c,d). On Day 3, females exhibited increased interactions with the new object compared to males (*F*_1, 56_ = 13.71, *p* < 0.001). A cycle/sex interaction was found for preference of the new object (*F*_1, 56_ = 5.95, *p* = 0.018), wherein LL/males exhibited reduced interactions with the new object compared to LD males (*p* < 0.001) but lighting condition had no effect on females (*p* = 0.52). Additionally, females had reduced interactions with the novel object compared to males in LD (*p* = 0.008), but not LL (*p* = 0.99) (Figures 4e,f).

## Discussion

This study uncovered several differences in the way consumption of a D1R agonist, SKF-38393 (DA), modulated behavior in mice under LL as well as sex differences in fluid consumption patterns. First, female mice consumed more DA compared to males, wherein males decreased their DA consumption, while females increased or had no change in their consumption over the course of the experiment. While we cannot definitely state that female mice exhibit increased DA preference compared to males because water consumption was also increased, other studies investigating drugs of abuse, including alcohol (Gelineau et al., 2017; Sneddon et al., 2019), cocaine (Russo et al., 2003; Roth and Carroll, 2004), and opiates (Hammerslag et al., 2021), illustrate an overall trend of increased drug intake in both binge-like and chronic consumption patterns in female rodents compared to males. There is also evidence to support that women also exhibit increased drug intake and addiction phenotypes compared to men (as reviewed by Becker and Hu, 2008; Little and Kosten, 2023). The estrous cycle is a potential influence on the behavioral difference seen between the males and females in this study, although it is worth noting that estrous phases were not measured in this study. Previous studies have illustrated connections among dopamine levels and altering phases on the mouse estrous cycle (Dazzi et al., 2007; Vandegrift et al., 2017). Differences in reward-seeking behavior exists in rodents in different phases of the estrous cycle and that progesterone attenuates, while estradiol can promote, drug-seeking behaviors (Giacometti et al., 2022; Hilz and Lee, 2023). While concentrations of DA and changes in inhibition might vary across studies, it is suggested that the change in ovarian hormones may play a role in the dopamine release and uptake in rodent models. Despite their reduced intake of DA compared to females, male mice still exhibited similar behavioral responses as females when consuming DA, indicating that the lower dosing of DA in males was still effective in attenuating some of the behavioral changes typically seen under LL.

In this study, LL induced anhedonia (reduced sucrose preference) in male mice but not female mice, and SKF-38393 consumption attenuated the depressive-like effects of LL in males. Other studies reported reduced sucrose and saccharine preference in male rodents housed under LL (Fonken et al., 2009; Tapia-Osorio et al., 2013; Tchekalarova et al., 2018; Capri et al., 2019; Yang et al., 2021), bright light-at-night (Li et al., 2024b), or dim light-at-night (Fonken et al., 2012; Bedrosian et al., 2013; Gutiérrez-Pérez et al., 2023). Additionally, a human study also showed increased depression and anxiety in humans who are exposed to light-at-night (Burns et al., 2023). Historically, SKF-38393 administration has been shown to alleviate depressive-like behaviors in rodents (Horita et al., 1991; D’Aquila et al., 1994), which has been corroborated by other D1R agonists (Desormeaux et al., 2020). Additionally, consumption of DA alleviated the LL-induced anxiety-like behaviors in both male and female mice, manifesting in reduced dark zone time and longer latency to first dark zone entry in the light–dark box. Other studies using housed under LL (Capri et al., 2019; Zhou et al., 2018; Yang et al., 2021) and other forms of bright light-at-night (Li et al., 2024a), reported increased anxiety, similar to what is found in humans (as reviewed by Tancredi et al., 2022). Alterations in D1R and DA signaling can induce anxiety within humans and animal models (Zweifel et al., 2011; Berry et al., 2019; Beyer et al., 2021) and treatment with D1R agonists, including SKF-38393, can reduce anxiety-like behaviors in rodents (Chan et al., 2017). In summary, these findings highlight the effects of LL on anxiety-like and depressive-like behaviors, the anxiolytic and antidepressant potential of SKF-38393 consumption, and the broader relevance of DA signaling and treatments in mitigating behavioral changes.

This study corroborates the previous results of novelty-induced hyperactivity in LL (Marin et al., 2015; Capri et al., 2019; Medeiros Contini and Seggio, 2023; Schröder et al., 2023), manifesting in increased locomotor activity within the open field. An open question that remains is: what behavior is this open field hyperactivity modeling? One possibility may be due to the animals being tested during their inactive period (ZT/CT 6), measuring increased arousal behavior. If the animals were tested during another part of the circadian cycle, such as during the night or in the dark, when arousal levels are generally higher in nocturnal animals, different open field results may have been found, as another study found (Richetto et al., 2019). Another potential model may be Attention-Deficit/Hyperactivity Disorder (ADHD), which manifests in lack of attention, increased impulsivity, and hyperactivity, and can be treated with dopamine agonists. Light-at-night is known to reduce inhibition and increase impulsivity in both humans and animal models (Byun et al., 2018; Cavalli et al., 2021; Meijdam et al., 2023). Other circadian disruptions, including shortened-day-cycles and simulated shift-work, can produce hyperactivity within the open field in rodents (McGowan and Coogan, 2013; Maroni et al., 2020). Connections exist between proper circadian signaling and ADHD—deficiencies in “core clock genes” including *period1* and *rev-erbα* lead to both hyperactivity and alterations to dopaminergic signaling in animal models (Huang et al., 2015; Otsuka et al., 2022). Humans with ADHD are also known to have mutations within the *period* genes and altered temporal regulation of *period* genes transcription (Wang et al., 2020; Faltraco et al., 2021). Both mouse and rat models of ADHD exhibit increased novelty-induced locomotor activity and impulsivity, which can be ameliorated by dopamine agonists (Kamimura et al., 2001; Majdak et al., 2016; Bouchatta et al., 2018). As such, the amelioration of the open field hyperactivity seen in the DA-consuming mice in this study may be an emerging model of ADHD-like behaviors.

We found several sex differences in the behavioral response of DA-consumption in both LD and LL, notably the ineffectiveness of DA to ameliorate the anhedonia of LL in female mice. It is somewhat surprising that female mice seemed to be resistant to the depressive effects of LL, given that females tend to be more sensitive to depressive effects of stressors and have higher rates of depression compared to males (Hasbi et al., 2020; Kropp and Hodes, 2023). The first possibility to this discrepancy may be due to the method of light-at-night used in the aforementioned studies, which used dim light-at-night and found anhedonia, and the current study which used chronic bright light and found no anhedonia; as such, the brightness of the light may lead to differences in behavioral responses. Female mice exhibit reduced depressive-like behaviors when tested under bright light compared to males as well as compared to females tested in the dark phase (Huynh et al., 2011). Sex differences also exist in the behavioral responses to anxiety and depression assays regarding the time-of-day when the tests are administered, wherein female mice exhibit increased anxiety-and depressive-like behaviors when tested in the dark phase compared to males (Verma et al., 2010); if we assayed the behavior during a different circadian or estrus cycle time, differing results may have been found. Female mice also show increased dopaminergic release following exposure to tests of depression and stressors compared to males (Dalla et al., 2010; Kokras et al., 2018). The bright light and the increased dopamine release may provide a countermeasure for the observance of anhedonia in females, although it is worth noting that DA levels, DA receptors, nor DA release were not measured in this study. Second, the removal of the DA for the sucrose preference test may have led to a mild form of withdrawal, which could alter the behavioral response in female mice. Generally, female rodents and women tend to be more susceptible to withdrawal symptoms and abstinence from a drug compared to males (Becker and Koob, 2016; Nicolas et al., 2022). These differences may explain the lack of depressive-like effects in water consuming female mice, as well as the reduced sucrose preference compared to males when consuming DA, under bright LL. Research also suggests that, in adult rodents, females tend to show an increase in locomotor activity when given injections of D1R agonists, including SKF-38393, compared to males (Díaz-Véliz et al., 1999; Heijtz et al., 2002; Schindler and Carmona, 2002). This increase in activity is modulated by the estrous cycle as well, wherein high-dose SKF-38393 injections produce increased locomotor activity within female rodents in diestrus compared to estrus (Díaz-Véliz et al., 1999). This result may partially explain why LD/F/DA animals exhibited increased distance traveled compared to the water controls and males. These findings highlight the importance of considering sex differences in behavioral responses to D1R agonists, but future work is needed to further elucidate the underlying mechanisms of how D1Rs influence anxiety, depression, and locomotion between males and females.

Although SKF-38393 is known to activate D1Rs, SKF-38393 also binds to D5Rs, so the question remains whether the behavioral effects of SKF-38393 on LL are at least partially mediated by D5Rs, rather than predominately D1Rs. While numerous behavioral abnormalities exist in rodent models with reduced D1Rs including hyperactivity, fewer exist in D5R knockouts, which may imply that the behavioral effects of DA-consumption in this study may be mostly due to D1R-specific pathways (Karlsson et al., 2008; Beyer et al., 2021; Sasamori et al., 2022). Interestingly, the effects of DR1 agonist injections (including SKF-38393) vs. oral consumption in this study on behavioral responses is somewhat contradictory and may depend upon dosing, the specific agonist/antagonist, and the injection site. For example, higher doses of SKF-38393 (> 2.5 mg/kg) produce increases to locomotion, impulsivity, and memory (Loos et al., 2010; Lejeune et al., 2013; Zhu et al., 2017), while low doses (including this study) have been shown to reduce impulsivity and hyperactivity, improve attention and vigilance, and have smaller effects on memory (Granon et al., 2000; Barnes et al., 2012; Abdulkader and Gigg, 2024). These smaller effects on memory due to low dosing may be why dopamine had little effects on memory within the object recognition test. Indeed, oral consumption of SKF-38393 has no effect on long-term potentiation by itself, although it can increase long-term potentiation if paired with nicotine and cocaine (Huang et al., 2014). Additionally, higher doses with injections tend to produce anxiogenic effects and in the case of antagonists can produce either anxiogenic or anxiolytic effects; different results on behavior also exist if the D1R is co-administered with other drugs, such as cocaine or nicotine (summarized by Zarrindast and Khakpai, 2015). By drinking the SKF-38393, it opens up the agonist to “first-pass metabolism,” which occurs in D1R agonists (Wu et al., 2005), potentially leading to dose-dependent biphasic responses. To our knowledge, the vast majority of studies have investigated the effects of injections of D1R agonists on behavior, rather than using oral consumption. Additional research is needed to determine how gut and liver metabolism can alter how D1R agonist oral consumption manifest behaviorally.

## Conclusion

In conclusion, this study highlights the complex interplay among LL, dopamine agonism, and sex differences in modulating behavioral outcomes in mice. LL induced anxiety-like, hyperactive, and depressive-like behaviors, many of which were mitigated by the consumption of DA. Importantly, while DA attenuated anxiety and hyperactivity in both sexes, its effects on anhedonia was sex-specific, with males showing greater benefit. Moreover, this study supports prior findings on the anxiogenic and hyperactivity-inducing effects of LL, while providing new insights into how DA consumption modulates these behaviors, potentially offering a model for conditions such as ADHD. Future research should explore the mechanistic roles of D1R and D5R pathways, dose-dependent effects of DA, and the influence of circadian timing on behavioral outcomes to deepen our understanding of these interactions and their implications for translational research in mood disorders.

## Data Availability

The raw data supporting the conclusions of this article will be made available by the authors, without undue reservation.
